# The Hepatoprotective Effect of Two Date Palm Fruit Cultivars’ Extracts: Green Optimization of the Extraction Process

**DOI:** 10.3390/foods12061229

**Published:** 2023-03-13

**Authors:** Nashi K. Alqahtani, Hisham A. Mohamed, Mahmoud E. Moawad, Nancy S. Younis, Maged E. Mohamed

**Affiliations:** 1 Department of Food and Nutrition Sciences, Faculty of Agriculture and Food Sciences, King Faisal University, P.O. Box 400, Al-Ahsa 31982, Saudi Arabia; 2 Date Palm Research Center of Excellence, King Faisal University, P.O. Box 400, Al-Ahsa 31982, Saudi Arabia; 3 Agricultural Research Center, Ministry of Agricultural, Giza 58312, Egypt; 4 Pathology Department, National Cancer Institute, Cairo University, Giza 12613, Egypt; 5 Pharmaceutical Science Department, Faculty of Clinical Pharmacy, King Faisal University, P.O. Box 400, Al-Ahsa 31982, Saudi Arabia; 6 Zagazig University Hospitals, Zagazig University, Zagazig 44519, Egypt; 7 Department of Pharmacognosy, College of Pharmacy, Zagazig University, Zagazig 44519, Egypt

**Keywords:** apoptosis, carbon tetrachloride, date palm, inflammation, liver damage, response surface methodology, Anbara, Reziz, water bath-assisted extraction

## Abstract

Date palm fruit (*Phoenix dactylifera*: Arecaceae) is rich in essential nutrients and possesses several pharmacological and medicinal activities. The current study aimed to optimize a water bath-assisted extraction method for two cultivars of date palm fruits, Anbara (An) and Reziz (Rz), and investigated the protective effect of the optimized date palm fruit extract against CCl_4_-induced liver toxicity in relation to oxidative stress, inflammation, apoptosis, and DNA integrity. The optimization process of two date palm fruit cultivars was applied, using response surface methodology through adjusting three “factors”; time, temperature, and rotation, to allow maximum contents of total phenolic (TPC), total flavonoid (TFC), reducing power (FRAP) and scavenging activity (ABTS) of the extract “responses”. Extraction factors’ application significantly enhanced TPC, TFC, FRAP, and ABTS responses by 1.30, 1.23, 3.03, and 2.06-fold, respectively in An and 2.18, 1.71, 1.11, and 2.62-fold, respectively in Rz, in relation to the convectional water extraction. Furthermore, co-administered CCl_4_ with An or Rz optimized extracts enhanced body weight gain, amended hepatic architecture, and diminished collagen fiber accumulation. Furthermore, An or Rz extracts reduced liver enzymes, hydroxyproline, alpha-fetoprotein (AFP), MDA, inflammatory cytokine (TNF-α, NF-κB) levels, and DNA fragmentation, while increasing deteriorated adiponectin (ADP) and antioxidant enzyme (GSH, GPX, NO, and IFN-γ) levels, relative to CCl_4_-administered animals. The protective effects of An or Rz-optimized extracts were also evidenced by suppressing hepatic fibrosis and improving liver function and structure via modulating oxidative stress, inflammation, and apoptosis, in CCl_4_-induced hepatic damage. Hence, the optimized extraction process for the two date palm fruits resulted in extracts which are rich in phenolic and flavonoid contents and with an elevated antioxidant power. The presence of these rich extracts could help to explain their proven hepatoprotective activity against CCl_4_-induced liver toxicity.

## 1. Introduction

Date palm fruit (*Phoenix dactylifera*: Arecaceae) is cultivated throughout the Middle East and other regions worldwide, including Central and South America, Europe, India, and the USA [1]. Date palm fruits are rich in numerous essential nutrients and are one of the most commonly consumed fruits in the Middle East and North Africa [2]. Several medicinal and pharmacological actions have been described for date palm fruits, including antioxidant [3], anti-inflammatory [4], gastroprotective [5], nephroprotective [6], anticancer [7], and immunostimulant [8] activities. Furthermore, a randomized controlled human study investigated the impact of palm date fruit consumption on microbiota growth and large intestinal health and concluded that fruit consumption might reduce colon cancer risk without inducing changes in the microbiota [9]. Another randomized controlled trial explored the effects of daily low-dose date palm fruit consumption on glycemic control, lipid profile, and quality of life in pre-and type-two diabetic patients. The study suggested that date palm fruits could benefit lipid profiles because of their high polyphenolic content [10]. Regarding its hepatic action, date extract ameliorated hepatic injury induced by cisplatin [11], gentamicin [12], and mercury [13], among others.

The consumption of date palm fruits can be as fresh or dried fruits; nevertheless, the potential to process these fruits into commercial products such as extracts, syrups, and concentrates is high and gives an added value to the fruit [14]. To process date palm fruits, the fruit should normally be extracted. Extraction methods vary; however, they can be categorized into two main categories, conventional and unconventional. Conventional extraction methodologies such as maceration, percolation, and reflux suffer the use of a large volume of solvents, long application time, low extractive values, and the utilization of non-eco-friendly solvents [15].

“Green extraction” is a term that describes the design of extraction processes that could overcome part of the disadvantages of conventional extraction methods. Green extraction methods permit the moderation of energy consumption, the use of green solvents, and the assurance of high-quality and safe extracts [16]. Using water as a green solvent has tremendous benefits, including inexpensiveness and being environmentally benign. As a green solvent, water is also non-flammable and nontoxic, providing opportunities for clean processing and pollution prevention. However, water is a polar solvent with impaired solubility toward the lyophilic matrices, a problem that can be overcome by manipulating the physical conditions of the extraction process [17]. Examples of physical conditions that could be changed are the temperature, the contact time, and the use of physical aids such as microwaves, ultrasonic sound waves, or rotation. Water bath-assisted extraction (WAE) is a method of extraction that gathers three physical aids with water as a solvent: temperature, time, and rotation. This method is implemented through shaking incubators, which allow temperature, time, and shaking (rotation) control. This method is commonly used to extract herbal and natural products [18,19], although current research has rarely implemented this method in date palm fruit extraction [20].

Carbon tetrachloride (CCl_4_) is a potent hepatotoxic compound that is widely applied for the initiation of chemically-induced hepatic damage in animal models involving oxidative stress and inflammation [21,22]. The harmful effect of CCl_4_ can be ascribed to the conversion of CCl_4_ to highly toxic free radicals (the trichloromethyl and the trichloromethyl peroxyl radicals), with consequent oxidative stress and inflammation [23]. Furthermore, these toxic radicals stimulate lipid peroxidation in structures rich in phospholipids, such as mitochondria and endoplasmic reticulum [24]. Furthermore, these free radicals prompt many catabolic processes, such as apoptosis, necrosis, and autophagy [25]. Hence, this hepatotoxic animal model was used to study the effect of several naturally occurring antioxidants, such as plants or plant-derived phytochemicals [26,27,28,29]. 

In the current study, the main aim was to apply water, as a green solvent, in extracting two cultivars of date palm fruits by developing a WAE method. The study investigates various factors affecting the extractions of the phenolic compounds and flavonoids as leading indicators for the antioxidant activity of the extract. Furthermore, the study extended to scrutinizing the protective effect of date palm fruit extract against CCl_4_-induced liver damage, and the development of fibrosis was studied in relation to the interdependence of oxidative stress, inflammation, apoptosis, and DNA integrity. 

## 2. Materials and Methods

### 2.1. Date Palm Fruits

Two cultivars of date palm fruits, Anbara (An) and Reziz (Rz), were freshly obtained (1 kg) from original date palm orchards at the Date Palm Research Center of Excellence, King Faisal University, Al-Ahsa, KSA. The fruits were at the complete ripening stage (Tamar) and were randomly selected in a medium weight, without any physical, insect, or fungal infection damage. The fruits were collected according to the scale recorded by Abdulhadi [30] and then authenticated by the plant taxonomist at the Date Palm Research Center of Excellence, KFU, KSA. Each fruit cultivar was put in sterilized paper sample bags and deposited in the refrigerator at 5 °C until experimental use. 

### 2.2. Conventional Extraction of the Date Palm Fruit Cultivars via Ethanol

Ethanolic water extractions were carried out using the procedure described by Siddeeg et al. [31]. The 100 g date palm fruits from both cultivars, An and Rz, were pitted, sliced, and then placed in 600 mL ethanol: water (4:1 (*v*/*v*)) or distilled water for 12 h. The extracts were filtered and centrifuged (2500× *g* for 10 min). The extracts were evaporated under reduced pressure, and residues were kept at 4 °C until use.

### 2.3. Green Extraction of Date Palm Fruits Using Water Bath-Assisted Extraction (WAE)

#### 2.3.1. The Experimental Design

Design-Expert statistical software (Stat-Ease Inc., Minneapolis, software version 11.1.2.0, MN 55413 USA) was used for the WAE’s experimental design and statistical analysis, using a randomized central composite design as a type of response surface analysis. The design included the implementation of four “factors”, three of which were continuous: extraction “temperature” (included three levels, 30, 45, and 60 °C); extraction “time” (included three levels, 20, 40, 60 min); extraction “rotation” (included three levels, 50, 100 and 150 rpm) (Table 1). The fourth factor was the category factor, the type of date palm fruit “cultivar” (included two categories, An and Rz). The experimental design model related all the “factors” with four different “responses”: total phenolic content (TPC), total flavonoid content (TFC), ferric reducing antioxidant power (FRAP), and the ability of free radical scavenging using ABTS (ABTS) (Table 1). Statistical analyses of these data were carried out using analysis of variance (ANOVA), and the quadratic model was the best fit, with *p*-values for all responses of less than 0.0005 and R^2^ and adjusted R^2^ values scoring no less than 0.85 and 0.89, respectively. Three-dimensional surface plots, through the response surface methodology (RSM) model, were performed using the same software as above (Design-Expert). Optimization was computed using the desirability methods, with all calculated values of desirability varying from 0.5 to 0.6. The goal criteria in numerical optimization were set to “minimize” in the case of factors (time and temperature) and to “maximize” in the case of responses (TPC, TFC, FRAP, and ABTS). The generated statistical models were all validated by comparing experimental and predicted response values.

#### 2.3.2. Preparation of Date Palm Fruits for WAE

The flesh (pericarp) of the previously mentioned date palm fruit cultivars was sliced (approximate dimensions 1.0 × 1.0 × 0.3 cm), and then from each date palm cultivar, 100 g was mixed with deionized distilled water (200 mL) in a ratio of 1:2 (*w*/*v*).

#### 2.3.3. Water Bath-Assisted Extraction (WAE) Method

A digitally controlled water bath with the orbital vibrator (Lab Companion Reciprocal Shaking Water Baths, Jeio Tech) was used in the WAE implementation. The sample was deposited in a glass flask (250 mL) and partially dipped into the water bath, with the flasks’ bottoms approximately 2.0 cm above the bathtub. The water bath was adjusted to the required temperature (Factor 2, Table 1), and then the rotation was adjusted to the required value (Factor 1, Table 1). Finally, the time was adjusted as required (Factor 3, Table 1), and the experiment was allowed to run. Then, the flask contents were transferred to falcon tubes (50 mL) and centrifuged at 2500× *g* for 10 min at 5.0 °C. The supernatant was decanted, filtered into a clean test tube, and then stored at 4 °C. All samples were diluted with distilled water (1:20) before assessing all dependent variables (responses), as mentioned below.

### 2.4. Determination of Experimental “Response” Parameters

#### 2.4.1. Total Phenolic Content (TPC) Determination

The Folin–Ciocalteau colorimetric assay [32] was used to determine the TPC in the date palm extracts using gallic acid (product number G7384, Merck, Darmstadt, Germany) as a standard. A total of 30 μL of the diluted sample or gallic acid serial dilution (25, 50, 75, and 100 g/mL in methanol) was added to 150 μL of (1:10) dilution of Folin–Ciocalteau reagent (product number 1.09001, Merck, Darmstadt, Germany) in water. The contents were mixed for 5 min, and then 6% Na_2_CO_3_ (120 μL) was added to obtain a blue-colored solution after incubating for 30 min at 40 °C. Absorbance was determined at 765 nm using a microplate reader (Shimadzu, Kyoto, Japan). Triplicate samples were utilized for each determination, and TPC values were calculated as gallic acid equivalents (GAE: mg/100 g fresh sample). 

#### 2.4.2. Total Flavonoid Content (TFC) Determination

An aluminum chloride colorimetric assay [33,34] was applied to determine TFC using quercetin (standard, Q4951, Merck, Darmstadt, Germany) as a standard. A total of 40 μL of the diluted samples or quercetin serial dilution (0.25, 0.5, 0.75, and 1.0 mg/mL) was diluted into 100 μL with distilled water, before sodium nitrate solution (5%, 5 μL) was added to the solution and incubated for 5 min. AlCl_3_ solution (10%, 10 μL) was added, and the solution was left for 6 min, after which NaOH (1 M, 50 μL) and distilled water (100 μL) were added. The absorbance was determined immediately at 510 nm utilizing a microplate reader (Shimadzu, Kyoto, Japan). Triplicate samples were used for each measure, and TFC values were calculated as quercetin equivalents (QE: mg/100 g fresh sample). 

#### 2.4.3. Ferric Reducing Antioxidant Power Assay (FRAP)

The reduction of the ferric 2,4,6-tripyridyl-s-triazine complex (Fe^3+^-TPTZ) to its ferrous form (Fe^2+^-TPTZ) was implemented [35] as an indicator of the reducing power of the date palm extracts using L-ascorbic acid (standard, A92902, Merck, Darmstadt, Germany) as a standard. Acetate buffer (10 mL, 300 mmol/L, pH = 3.6), TPTZ solution (1.0 mL 10 mmol/L, product number T1253, Merck, Darmstadt, Germany), and FeCl_3_ (1.0 mL 20 mmol/L) were mixed in 40 mmol/L HCl to prepare the working solution. An aliquot of 20 μL of the sample, ethanol (blank), or L-Ascorbic acid was added to the working solution (180 μL) for 15 min at 37 °C. Absorbance was determined at 593 nm using a microplate reader (Shimadzu, Kyoto, Japan). The results were expressed as ascorbic acid equivalents (AE: mmol/100 g fresh sample).

#### 2.4.4. Radical-Scavenging Antioxidant Ability by ABTS

The scavenging capability of the date palm fruit extracts was determined using ABTS (2,2′-azinobis-3-ethylbenothiazoline-6-sulphonic acid-diammonium salt, ABTS, A9941, Merck, Darmstadt, Germany) radicals as an indicator for the antioxidant power of the extract [36] using L-Ascorbic acid (A92902, Merck, Darmstadt, Germany) as a standard. ABTS solution (10 mL, 7 mM) was mixed with K_2_S_2_O_8_ (10 mL, 2.45 mM) solution and maintained in the dark (12–16 h, room temperature). The ABTS/K_2_S_2_O_8_ solution was adjusted to 0.7 ± 0.02 at 734 nm with ethanol utilizing a spectrophotometer (Thermo Spectronic Genesys 20, T165762, 4001/4) just before application. The adjusted ABTS/K_2_S_2_O_8_ solution (1.95 mL) was added to the sample or L-Ascorbic acid (0.5 mL) solution, vortexed, and then incubated in the dark (30 °C for 5 min). Absorbance was determined (734 nm) against methanol (blank) using a microplate reader (Shimadzu, Kyoto, Japan). The results were calculated as mmol ascorbic equivalents/100 g fresh sample. The radical scavenging assays of all samples were presented as a percentage inhibition of absorbance (percentage of scavenging ABTS radicals) using the following equation:

Inhibition (%) or ABTS scavenging effect (%) = (A0 − A1/A0) × 100(1)

where A0 is the control absorbance and A1 is the absorbance of the sample. The control used was 100% distilled water. 

### 2.5. The Hepatoprotective Activity Assessment of Date Palm Fruit Cultivars

#### 2.5.1. Animals and Ethical Approval

Male Wistar rats (weight: 110 ± 10 g, age: 7–8 weeks old) were obtained from the animal house facility at King Saud University. Animals were kept in the laboratory under constant temperature (24 ± 3 °C), under a dark–light cycle (12/12 h) at least one week before and throughout the experimental work, and maintained on a standard pellet and allowed free access to water. 

The animal procedures followed the ARRIVE guidelines. The Institutional Animal Care and Use Committee approved the experimental protocol of King Faisal University (KFU-REC-2022-DEC-ETHICS400). All the experiments were executed in harmony with the relevant procedures and regulations of the Ethical Conduct for the Use of Animals in Research at King Faisal University. 

#### 2.5.2. Extraction of Date Palm Fruits for In Vivo Experiments

The two cultivars of date palm fruit, An and Rz, were extracted according to the optimized WAE developed above using the optimized values of the “factors”, time, temperature, and rotation to produce the maximum TPC, TFC, FRAP, and ABTS activities (Table 1).

#### 2.5.3. Experimental Design

Rats were randomly allocated into six groups (*n* = 8); the control group, in which normal rats were administered olive oil intraperitoneally twice weekly for 16 weeks; the An control group, in which rats were administered An extract (100 mg/kg; orally, daily) for 16 weeks [37]; the Rz control group, in which animals were given Rz extract (100 mg/kg; orally, daily) for 16 weeks; the carbon tetrachloride group (CCl_4_), in which animals were injected intraperitoneally with CCl_4_ (2 mL/kg) twice weekly for 16 weeks to induce liver injury [28]; the CCl_4_ + An and CCl_4_ + Rz groups, in which rats were administered either An or Rz extracts (100 mg/kg; orally, daily), respectively, with CCl_4_ (2 mL/kg, IP) in the same regimen for 16 weeks. CCl_4_ was dissolved in 1.0 mL of olive oil, as mentioned elsewhere [38].

#### 2.5.4. General Health and Body Weight Changes

Signs of the animals’ good health were observed, including the animal consumption of diet (appetite), the animal’s physical activity, the color of urine, stool condition, the condition of fur and skin, and the mortality rate. The initial (at zero time) and final (before animal scarification) body weights were measured, and ∆ change in body weight was calculated using the following formula:

Δ Change in body weight = ((final body weight − initial body weight)/initial body weight) × 100

#### 2.5.5. Collection of Blood Samples and Hepatic Tissues

At the end of the experiment, rats were euthanized with isoflurane (3%), and the blood samples were collected via cardiac puncture. The obtained blood was centrifuged for 20 min at 4000 rpm/min to attain the serum, which was stored until further biochemical analysis at −20 °C. The livers of the euthanized rats were dissected out on an ice bed and divided into two parts; the first part was used for the antioxidant parameters’ investigation, while the second part was washed in saline and immediately fixed into 10% neutral buffer formalin for the histopathological examination.

#### 2.5.6. Liver Toxicity Evaluation

Histological examination (hematoxylin and eosin (H&E) and Masson’s trichrome (MT)) and hydroxyproline content determination were performed to evaluate CCl_4_ hepatic toxicity.

##### Histopathological Examinations

Following fixation of the liver samples in 10% formalin overnight, samples were washed and dehydrated. Tissue specimens were cleared in xylene for 2–4 h to remove the alcohol and embedded in paraffin wax to form blocks. The paraffin blocks were sectioned at 3.0-micron thickness by slide microtome. The obtained tissue sections were collected on the glass slides and stained by H&E stain to be examined using the light microscope. 

Another set of paraffin-embedded liver tissue sections (5 to 7 mm) was stained with Masson’s trichrome (MT) to measure hepatic fibrosis. The sections were examined under a light microscope (Leica microscope, Berlin, Germany) and photographed. The blue stain reflects the extent of hepatic fibrosis and is employed to identify collagen fibers [39]. Image analysis methods were performed using Image J software.

##### Determination of Hydroxyproline

Rat ELISA hydroxyproline kits (MBS017427) were purchased from MyBioSource (San Diego, CA, USA) and performed according to the manufacturer’s instructions. 

#### 2.5.7. Determination of Liver Function

Serum alanine aminotransferase (ALT), aspartate aminotransferase (AST) activities, alkaline phosphatase (ALP), gamma glutamyl transferase (γGT), and lactate dehydrogenase (LDH) were assayed spectrophotometrically via commercially available kits (Spectrum, Cairo, Egypt) following the manufacturer’s instructions.

#### 2.5.8. Enzyme-Linked Immunosorbent Assay Evaluations

Rat ELISA kits of alpha-fetoprotein (AFP, MBS267612), malondialdehyde (MDA, MBS738685), glutathione (GSH, MBS265966), glutathione peroxidase (GPX, MBS744364), nitric oxide (NO, MBS2604161), and adiponectin (ADP, MBS285758) were obtained from MyBioSource (San Diego, CA, USA) and accomplished in accordance with the manufacturer’s instructions. IFN gamma (IFN-γ, ab239425), nuclear factor-kappa B (NF-κB p65, ab176648), and tumor necrosis factor-alpha (TNF-α, ab100785) ELISA kits were procured from Abcam Inc. (Cambridge, UK).

#### 2.5.9. Apoptosis Assay via the Detection of DNA Fragmentation

DNA fragmentation is a characteristic sign of apoptotic cell death. It can be detected as a DNA laddering pattern using agarose gel electrophoresis [40]. Cells were washed with PBS and then lysed in cold lysis solution (5 mmol/L Tris, pH 7.4, 20 mmol/L EDTA, 0.5% Triton X-100) for 20 min. Cell lysates were centrifuged at 27,000× *g* for 15 min, and DNA was extracted from the aqueous phase with phenol: chloroform: isoamyl alcohol (25:24:1, *v*/*v*/*v*) containing 0.1% (*w*/*v*) hydroxyquinoline. DNA was precipitated with 0.3 mol/L of potassium acetate and two volumes of cold 100% (*v*/*v*) ethanol. Agarose gel (3% *w*/*v*) electrophoresis proceeded at 30 mA for 2 h, followed by UV fluorescence to determine the degree of apoptotic DNA fragmentation. A picture of the gel was taken via the gel documentation system (BioRad, Berkeley, CA, USA) [41]. 

### 2.6. Statistical Analysis

Data normality was verified using SPSS software, applying two tests: Kolmogorov–Smirnov and Shapiro–Wilk. Data were presented as mean ± SD. For multiple comparisons, one-way ANOVA was performed followed by Tukey–Kramer as a post hoc test. The 0.05 level of probability was used as the significance level. All statistical analyses were performed using Graph Pad software (version 8, San Diego, CA, USA). 

## 3. Results

### 3.1. Ethanol and Water Conventional Extraction Date Palm Fruits Cultivars

The two date palm fruit cultivars, An and Rz, were conventionally extracted using hydro-ethanol and distilled water, respectively. Other “factors” of the WAE were not applied, and the “time” factor was increased to 12 h, as traditionally applied in conventional extraction methods; the results are stated in Table 2. The results indicated the low power of water as a solvent of extraction related to hydro-ethanol. Water extracted only 29.6% and 25.1% of the TPC which could be extracted by hydro-ethanol in An and Rz cultivars, respectively. Similarly, water extraction represented 28.4% and 32.6% of the hydro-ethanol-extracted TFC in the four cultivars. The extract’s reducing and antioxidant capacity was diminished by applying water as a solvent. The FRAP assay values (representing the extract-reducing power) were lessened to 35.1% and 47.7% of the hydro-ethanol extract in the An and Rz cultivars. Comparably, the ABTS assay values (representing extract scavenging capability) were reduced to 29.8% and 27.9% of that of the hydro-ethanol extract in the same two cultivars, respectively.

### 3.2. Production of Date Palm Fruit Extract Applying the WAE Approach

Using solvents based on water, a green extraction technique was implemented to extract the two varieties of date palm fruits with the combination of temperature, extraction time, and rotation values as “factors”. These “factors” were chosen as they are a major influence on extracting phenolic compounds from date palm fruits, including flavonoids.

#### 3.2.1. Effect of WAE the Date Palm Fruit Varieties on TPC

**Figure 1:**
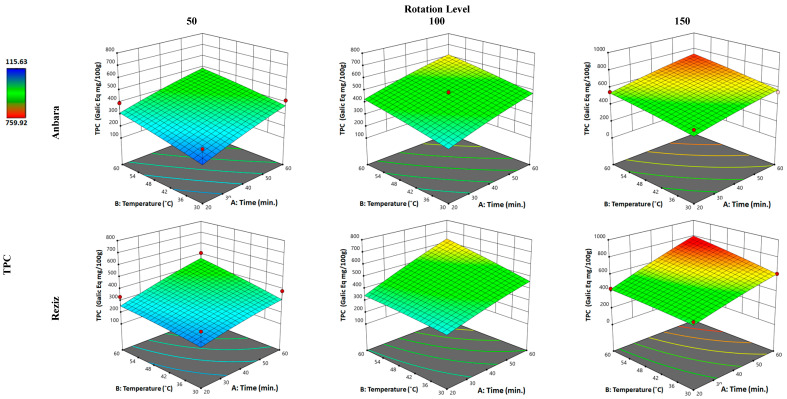
Three-dimensional response surface plot for “response” variable: (total phenolic content (TPC)), as a function of WAE “factors”: temperature, time, and rotation in two date palm fruits cultivars, Anbara and Reiziz.

Figure 1 illustrates the interactions between the three “factors” (temperature, time, and rotation) with TPC as a “response” for the two cultivars under investigation. For the An variety, applying the minimum in all “factors”, i.e., 20 min, 30 °C, and 50 rpm produced nearly no change in the amount of TPC extracted in relation to conventional water extraction (Table 1). Increasing the contact extraction time only (from 20 to 60 min) and applying the minimum of other “factors”, i.e., 30 °C and 50 rpm resulted in an increase of 1.57-fold from conventional water extraction (Table 1). Increasing both times (from 20 to 60 min) and the temperature (from 30 to 60 °C) produced a 1.89-fold increase, while applying the maximum of the three factors resulted in an elevation of 2.52-fold from conventional water extraction (Table 1). A similar status was observed with the Rz variety. Applying the minimum in all “factors”, i.e., 20 min, 30 °C, and 50 rpm produced a 1.6-fold change in the amount of TPC extracted in relation to conventional water extraction (Table 1). When the contact time was increased (from 20 to 60 min, applying the minimum of other “factors”, i.e., 30 °C and 50 rpm), the extracted TPC increased 2-fold from conventional water extraction (Table 1). Elevating both time and temperature to a maximum (60 min and 60 °C) augmented the TPC extracted by 2.7-fold; however, when all the “factors” were elevated to their maximum values, the extraction of TPC reached a 4-fold increase. The maximum extracted values of TPC applying WAE and using water as a solvent reached 74.9% and 96.4% from the extraction value of hydro-ethanol in the An and Rz varieties, respectively.

#### 3.2.2. Effect of WAE the Date Palm Fruit Varieties on TFC

**Figure 2:**
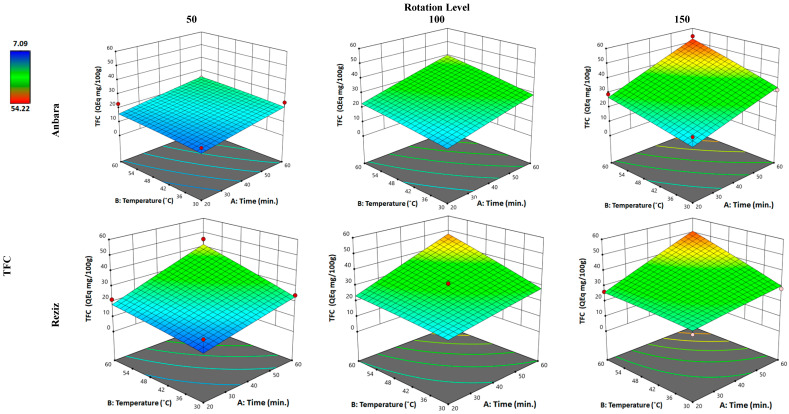
Three-dimensional response surface plot for the “response” variable: (total flavonoid content (TFC)), as a function of WAE “factors”: temperature, time, and rotation in two date palm fruits cultivars, Anbara and Reiziz.

The effect of the three factors (temperature, time, and rotation) on TFC extraction in the two date palm fruit cultivars, An and Rz, is discussed in Figure 2. Applying the minimum condition of WAE (20 min, 30 °C, and 50 rpm), the TFC extracted was reduced to nearly 0.83-fold of the conventional water extraction (Table 1) in the An cultivar. Increasing the extraction duration only (from 20 to 60 min) while applying the minimum of other “factors”, i.e., 30 °C and 50 rpm, resulted in an increase of 1.32-fold relative to conventional water extraction (Table 1). Increasing both time (from 20 to 60 min) and the temperature (from 30 to 60 °C) produced a 1.48-fold increase, while applying the maximum of the three factors resulted in an elevation of 2.9-fold relative to conventional water extraction (Table 1). Similarly, in the Rz variety, operating the minimum in all “factors”, i.e., 20 min, 30 °C, and 50 rpm, produced a minor increase in the amount of TFC extracted (1.1-fold) relative to conventional water extraction (Table 1). When the contact time was increased (from 20 to 60 min, applying the minimum of other responses, i.e., 30 °C and 50 rpm), the extracted TFC increased by 1.48-fold relative to conventional water extraction (Table 1). Elevating both time and temperature to a maximum (60 min and 60 °C) augmented the TFC extracted by 2.72-fold; however, when all the “factors” were elevated to their maximum values, the extraction of TFC did not increase (2.68-fold). The maximum extracted values of TFC applying WAE and using water as a solvent reached 85.7% and 92.0% compared with the extraction value of hydro-ethanol in the An and Rz varieties, respectively.

#### 3.2.3. Effect of WAE the Date Palm Fruit Varieties on FRAP

**Figure 3:**
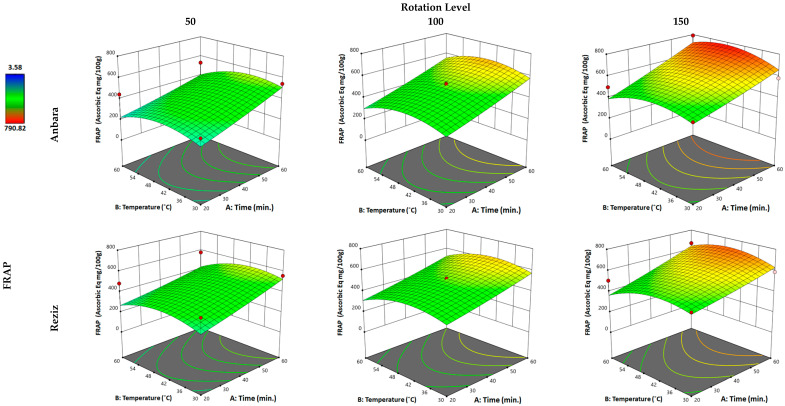
Three-dimensional response surface plot for the “response” variable: (ferric reducing antioxidant power assay (FRAP)), as a function of WAE “factors”: temperature, time, and rotation in two date palm fruits cultivars, Anbara and Reiziz.

Figure 3 states the influence of the three factors (temperature, time, and rotation) on FRAP for the two cultivars, An and Rz. For the An variety, utilizing the minimum in all “factors”, i.e., 20 min, 30 °C, and 50 rpm reduced the FRAP of the extract (0.93-fold) relative to conventional water extraction (Table 1). Increasing the duration only (from 20 to 60 min) and applying the minimum of other “factors”, i.e., 30 °C and 50 rpm resulted in an increase of 1.55-fold from conventional water extraction (Table 1). Increasing both the “time” (from 20 to 60 min) and the “temperature” (from 30 to 60 °C) factors produced a 1.66-fold increase while operating the maximum of the three factors resulted in an elevation of 2.25-fold relative to conventional water extraction (Table 1). For the Rz variety, applying the minimum in all “factors”, i.e., 20 min, 30 °C, and 50 rpm produced nearly no change in FRAP relative to conventional water extraction (Table 1). When the contact “time” was increased (from 20 to 60 min, applying the minimum of other “factors”, i.e., 30 °C and 50 rpm), the FRAP increased by 1.2-fold relative to conventional water extraction (Table 1). Increasing both time and temperature to a maximum (60 min and 60 °C) did not improve the FRAP (1.2-fold); however, when all the “factors” were elevated to their maximum values, the FRAP improved by 1.46-fold. The maximum extracted values of FRAP applying WAE and using water as a solvent reached 79.3% and 84.2% compared with the extraction value of hydro-ethanol in the An and Rz varieties, respectively.

#### 3.2.4. Effect of WAE the Date Palm Fruit Varieties on Scavenging Activity (ABTS Assay)

**Figure 4:**
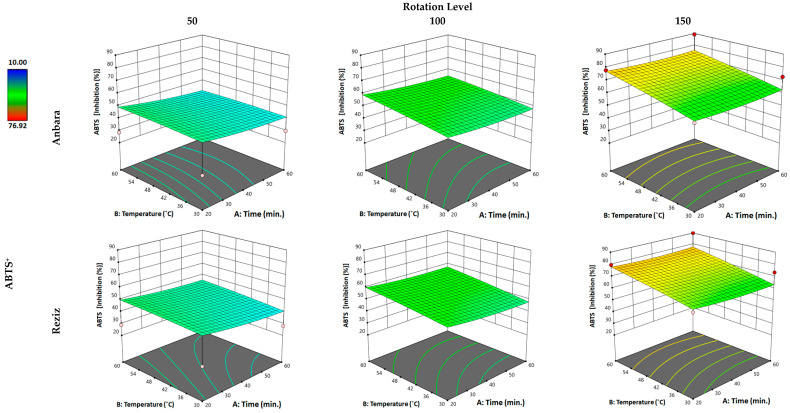
Three-dimensional response surface plot for the “response” variable: (radical-scavenging antioxidant ability by ABTS), as a function of WAE “factors”: temperature, time, and rotation in two date palm fruits cultivars, Anbara and Reiziz.

The effect of the temperature, time, and rotation on the scavenging activity (ABTS assay) for the two cultivars An and Rz is explained in Figure 4. For the An variety, using the minimum in all “factors”, i.e., 20 min, 30 °C, and 50 rpm produced no change in the scavenging activity (ABTS) of the extract related to conventional water extraction (Table 1). Increasing the duration only (from 20 to 60 min) and applying the minimum of other “factors”, i.e., 30 °C and 50 rpm resulted in an increase of 1.26-fold relative to conventional water extraction (Table 1). Elevating both the “time” (from 20 to 60 min) and the “temperature” (from 30 to 60 °C) factors produced a 1.49-fold increase, while operating the maximum of the three factors resulted in an elevation of 3.81-fold relative to conventional water extraction (Table 1). For the Rz variety, operating the minimum in all “factors”, i.e., 20 min, 30 °C, and 50 rpm produced a minor increase in the scavenging activity (ABTS) (1.14-fold) when related to conventional water extraction (Table 1). When the contact “time” was increased (from 20 to 60 min, applying the minimum of other “factors”, i.e., 30 °C and 50 rpm), the scavenging power (ABTS) increased 1.35-fold relative to conventional water extraction (Table 1). Increasing both time and temperature to a maximum (60 min and 60 °C) further improved the scavenging activity (ABTS) (1.75-fold), while—when all the “factors” were elevated to their maximum values—the scavenging power (ABTS) improved to a 4.3-fold increase. The maximum extracted values of scavenging power (ABTS) applying WAE and using water as a solvent reached 113% and 121% compared with the extraction value of hydro-ethanol in the An and Rz varieties, respectively.

#### 3.2.5. WAE Optimization

RSM models were performed through which the optimization of the WAE process was computed using the desirability method; the results are stated in Table 3. The calculated optimized extraction procedures were adjusted to gain the maximum of each response using the minimum of the factors. For the An cultivar, when the rotation was adjusted to 103 rpm, at a temperature of 30.00 °C, and after 20.44 min (time adjustment), a maximum of 344.322 (GAE: mg/100 g fresh sample) TPC, 22.340 (QE: mg/100 g fresh sample) TFC, 1061.692 (AE: mmol/100 g fresh sample) FRAP activity, and 48.173% ABTS scavenging effect percentage could be reached. These values corresponded to increases of 1.30, 1.23, 3.03, and 2.06-fold in the four responses, respectively, compared with conventional water extraction. In the Reziz cultivar, optimization suggested the use of 108 rpm with 38.70 °C and 36.62 min to generate a TPC of 4.11.295 (GAE: mg/100 g fresh sample), a TFC 28.090 (QE: mg/100 g fresh sample), a FRAP activity of 506.509 (AE: mmol/100 g fresh sample), and an ABTS scavenging effect of 54.388%. These values gave a fold-increase of 2.18, 1.71, 1.11, and 2.62 for the four responses compared with conventional water extraction.

### 3.3. The Hepatoprotective Activity of Date Palm Fruit Cultivars

#### 3.3.1. General Health and Body Weight Changes (%)

The normal, An, and Rz groups exhibited good overall health and normal weight gain compared with the CCl_4_ group. However, while animals were administered, CCl_4_ showed reduced activity and appetite, yellow urine, poor nutrition, mental fatigue, rough and dull fur, lifeless eyes, and symptoms of chronic diarrhea. Additionally, one rat died in the CCl_4_ group, while the remaining rats survived until the experiment’s end. As regards body weight, CCl_4_ animals exhibited significantly reduced body weight gain compared with the control group, whereas animals treated with An or Rz showed restored body weight gain relative to CCl_4_ animals, as shown in Table 4. 

#### 3.3.2. The Histological Studies

Morphologically, the rat livers in the CCl_4_ group appeared swollen, rough in texture, stiff, and dark in color, with many small nodules on the surface. However, the livers were ruddier in color and the edges were more regular in the normal, An, and Rz groups, and the groups administered CCl_4_ + An and CCl_4_ + Rz relative to those in the CCl_4_ group.

##### Histological Examination Using H&E

**Figure 5:**
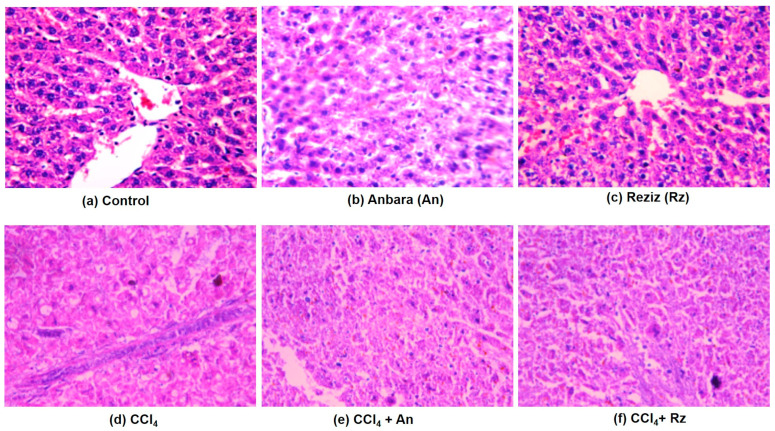
Histological examinations of the haptic sections’ staining by H&E of (**a**) normal, (**b**) An, and (**c**) Rz groups showing normal hepatic architectures, (**d**) CCl_4_ group exhibiting congested portal vein, leucocytic infiltration, activated Kupffer cells, congested central vein, and cytoplasmic vacuolation of hepatocytes with micro and macro-vesicular steatosis and fibrosis, (**e**) CCl_4_ + An revealing slight portal vessel congestion, and (**f**) CCl_4_ + Rz manifesting slight degeneration of hepatocytes infiltrated with leucocytes (H&E, ×400).

The control groups, including normal, An, and Rz, showed the normal architecture of hepatic lobules, as illustrated in Figure 5, whereas CCl_4_ resulted in the impaired structural organization of the hepatic lobules with loss of the characteristic cord-like arrangement, congested hepatic portal veins, and hepatocyte degeneration. Inflammatory leucocytic infiltrations were observed as well. The sinusoidal spaces were widened and contained activated Kupffer cells, the intrahepatic vessels were congested with blood, and intracellular hemorrhage appeared. The Kupffer cells displayed a noticeable activation, and an abnormally enlarged portal vein was observed. A considerable number of hepatocytes degenerated, while the others showed marked cytoplasmic vacuolation with micro and macro-vesicular steatosis and fibrosis. CCl_4_ + An animals showed improved hepatic architecture, despite the portal vein being congested and the presence of degenerated bile ducts. 

The hepatocytes, cytoplasm, and nuclei mainly showed normal histology, but blood sinusoids were dilated. CCl_4_ + Rz animals presented lower leucocytic infiltration and a precise central vein without any considerable congestion. However, Kupffer cells were still activated.

##### Histological Studies Using Masson’s Trichrome (MT) Stain

**Figure 6:**
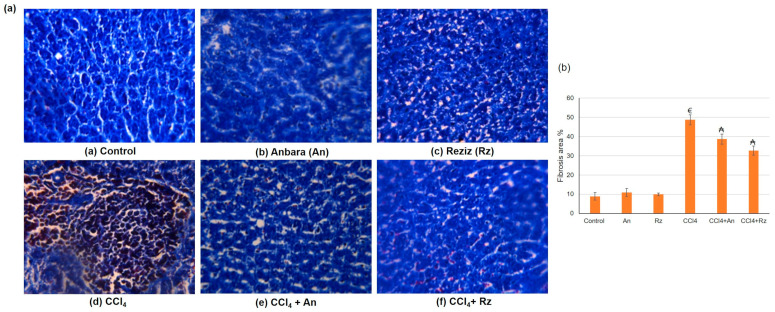
(**a**) Histological examination using Masson’s trichrome (MT) exposing that control, An, and Rz possess normal liver tissues showing no collagen precipitation, the CCl_4_ group present collagen fibers’ augmentation, CCl_4_ + An and CCl_4_ + Rz display reduced accumulation of collagen fibers and less fibrosis, and almost no steatosis was observed (×400) (**b**) Fibrosis area percentage. € describes a statistically significant correlation with the control, (₳) denotes a statistically significant correlation with the CCl_4_-induced hepatotoxicity group.

The control, An, and Rz groups displayed normal lobular architecture with central veins, uniform cells, neat rows of hepatic cords without degeneration, and the absence of fibrous tissue hyperplasia or pseudo-lobules. In addition, hepatic cell necrosis or collagen fibers were rarely observed (Figure 6). However, hepatic sections obtained from the CCl_4_ group displayed a markedly high number of inflammatory cell infiltrates, pronounced adipose degeneration of hepatocytes, extensive necrosis of hepatocytes around the lobule, and increased deposition of collagen fibers in hepatic lobules, all of which disturbed the lobular architecture and led to the formation of a pseudo-lobule that separated the lobules. On the other hand, histopathological examination of rat liver sections obtained from the CCl_4_ administered along with the date palm fruit extracts An and Rz groups revealed ameliorated adipose degeneration and necrosis of hepatocytes, reduced migration and infiltration of inflammatory cells, and declining collagen fiber deposition in hepatic lobules.

#### 3.3.3. Effect of An and Rz Extract Administration on Liver Enzymes in CCl_4_-Induced Hepatotoxicity

Liver enzymes were used to assess CCl_4_-induced hepatotoxicity. Table 5 shows the effect of administering CCl_4_ with An or Rz on liver enzymes. CCl_4_ administration caused a significant (*p*  <  0.001) escalation in the mean values of ALT, AST, ALP, γGT, and LDH, compared with those of the negative control group—indicating that CCl_4_ has induced hepatic injury—whereas consumption of CCl_4_ with An or Rz significantly (*p* <  0.001) decreased the mean values of the liver enzymes ALT, AST, ALP, GGT, and LDH compared with the CCl_4_-alone group. 

#### 3.3.4. Effect of An and Rz on Hydroxyproline Content in CCl_4_-Induced Hepatotoxicity

Hydroxyproline is an indicator of collagen deposition. During CCl_4_-induced hepatic injury, animals exhibited a significantly higher hydroxyproline content compared with the control, An, and Rz groups. Meanwhile, An and Rz administration and CCl_4_ resulted in a reduced level of hydroxyproline, which directly reflects a lesser amount of liver fibrosis, as shown in Table 6. 

#### 3.3.5. Effect of An and Rz on Serum Level of Alpha-Fetoprotein (AFP) in CCl_4_-Induced Hepatotoxicity

Serum AFP level was significantly elevated (*p* ≤ 0.05) in the CCl_4_ group compared with the normal, An, and Rz groups. However, supplementation of CCl_4_ with An or Rz resulted in a substantial decline (*p* ≤ 0.05) of the serum AFP level compared with that of the untreated CCl_4_ group, as shown in Table 6.

#### 3.3.6. Effect of An and Rz Administration on the Hepatic Content of Adiponectin (ADP) in CCl_4_-Induced Hepatotoxicity

Adiponectin has been demonstrated to have an anti-fibrotic action in the liver by blocking the activation of hepatic stellate cell-mediated adenosine monophosphate-activated protein kinase and peroxisome proliferator-activated receptor-alpha pathways, which in turn diminish the expression of pro-fibrotic genes [42]. Rats administered with CCl_4_ demonstrated a significant decrease in the hepatic content of ADP compared with control, An, and Rz groups. The hepatic content of ADP in animals administered with CCl_4_ alongside An or Rz exhibited a significant increase when compared with the CCl_4_ group (Table 6).

**Table 6:** Ameliorative effects of An or Rz administration on hydroxyproline, alpha-fetoprotein (AFP), and adiponectin (ADP) in different experimental groups during CCl_4_-induced hepatotoxicity.

Animal Group	Hydroxyproline (nmol/L)	α-Fetoprotein (AFP) (ng/mL)	Adiponectin (ADP) (ng/mL)
Control	23.43 ± 0.21	0.53 ± 0.25	8.07 ± 0.25
An	19.25 ± 0.21	0.54 ± 0.05	7.50 ± 0.14
Rz	22.67 ± 0.21	0.52 ± 0.03	7.70 ± 0.20
CCl_4_	75.23 ± 0.15 €	3.37 ± 0.42 €	3.87 ± 0.15 €
CCl_4_ + An	30.73 ± 0.21 ₳	1.45 ± 0.05 ₳	6.04 ± 0.05 ₳
CCl_4_ + Rz	39.33 ± 0.21 ₳	1.53 ± 0.03 ₳	5.57 ± 0.25 ₳

All data are enumerated as mean ± SE, (*n* = 6). (€) describes a statistically significant correlation with the control, (₳) denotes a statistically significant correlation with the CCl_4_-induced hepatotoxicity group using one-way ANOVA after Tukey’s post-hoc test (*p* < 0.05).

#### 3.3.7. Effect of An and Rz on Nitrosative and Oxidative Stress and Lipid Peroxidation in CCl_4_-Induced Hepatotoxicity

CCl_4_ injection induced a significant increase in MDA level (*p* ≤ 0.05) and significant declines (*p* ≤ 0.05) in the activity of GSH, GPX, and NO compared with those values in the control group and the An and Rz administered groups. However, co-administration of CCl_4_ with An or Rz displayed a significant decline in the MDA level (*p* ≤ 0.05) and a significant increase in the GSH, GPX, and NO activities compared with those in the untreated CCl_4_-induced hepatic toxicity, as shown in Table 7. An and Rz extracts increased MDA and NO levels relative to those in control animals.

#### 3.3.8. Effect of An and Rz on IFN-γ, TNF-α, and NF-κB in CCl_4_-Induced Hepatotoxicity

We further investigated the effect of date palm fruit extracts on inflammatory indicators such as IFN-γ, TNF-α, and NF-κB. NF-κB is a key upstream factor for various pro-inflammatory mediators. TNF-α and NF-κB were significantly elevated compared with the normal groups, whereas IFN-γ level was reduced in the CCl_4_-induced hepatotoxicity group. By contrast, TNF-α and NF-κB were diminished, whereas IFN-γ level was significantly increased in animals administered with CCl_4_ along with An or Rz extracts—indicating that IFN-γ plays a key role mainly in the early phase of liver injury induced by CCl_4_, as shown in Table 8. 

#### 3.3.9. The Effect of An and Rz on Apoptosis Using DNA Fragmentation Assays in CCl_4_-Induced Hepatotoxicity

**Figure 7:**
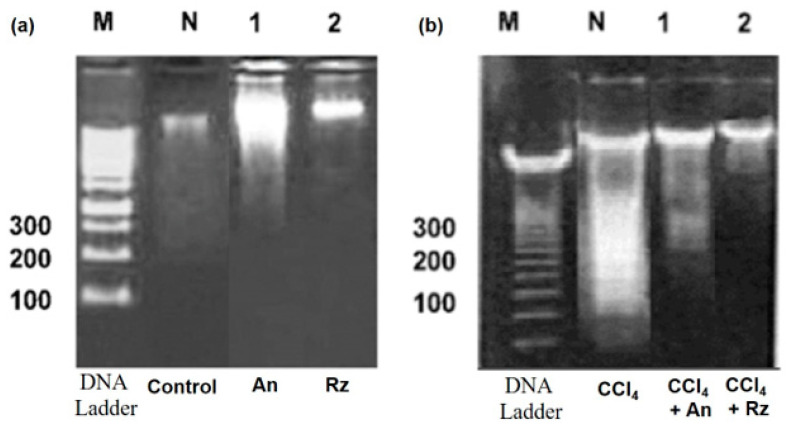
Gel electrophoresis of liver genomic DNA in different animal groups. (**a**) Lane M: DNA ladder, Lane N: normal liver control, Lane 1: An, Lane 2: Rz. (**b**) Lane M: DNA ladder, Lane T: CCl_4_, Lane 1: CCl_4_ + An, Lane 2: CCl_4_ + Rz.

DNA laddering is used as a basic feature for apoptosis. Casp-initiated DNase digests DNA at inter-nucleosomal linkers into distinct fragments of approximately 180 bp. The outcomes of the DNA fragmentation assay revealed a significant increase in the DNA laddering in the CCl_4_-challenged group. An and Rz were proven to be potent protective agents, as they attenuated the fragmentation percentage in the CCl_4_ + An and CCl_4_ + Rz groups compared with the CCl_4_-challenged group, as illustrated in Figure 7. 

## 4. Discussion

### 4.1. Optimization of An and Rz Cultivars of Date Palm Fruit Extraction Using WAE

Several extraction techniques are used nowadays to reduce the dependency on organic solvents and replace them with more eco- and environmental-friendly solvents, called “green extraction”. These techniques apply state-of-the-art technology to allow the use of less or no organic solvent and increase the yield of the medicinally or pharmacologically active phytochemicals [43]. Moreover, green extraction methods are applied to reduce the disadvantages associated with conventional extraction methods, such as the requirement to use large volumes of solvent and long extraction durations. Most green extraction methods utilize water as a solvent of extraction due to its many advantages; however, the use of water alone does not allow efficient extraction as water is a polar solvent with specific and low extractive powers, especially for lipophilic compounds [44]. Accordingly, the use of water as a green solvent is usually accompanied by a physical aid such as a microwave, rotatory water path, ultrasonics, etc. 

Date palm fruits are known for their therapeutic and pharmacological activities due to the presence of several classes of active phytochemicals, such as phenolic compounds and flavonoids. Many of these compounds are slightly soluble in water, particularly those of an aglycone nature (i.e., non-glycosides) [43,44]. Consequently, the extraction techniques should be directed to extract as much as possible of these valuable phytochemicals.

In this study, the application of water only as an extraction solvent, even with increasing the time to 12 h, afforded low extractive values for TPC (29.6% and 23.9%) and TFC (28.8 and 32.7%) when compared with organic solvent (ethanol) extraction for the palm fruit cultivars An and Rz, respectively. Furthermore, extraction with water, without any physical aids, produced an extract with a low antioxidant power; the FRAP values were 35.1% and 48.6%, and the ABTS values were 29.9% and 27.9% of the ethanol extract values for each cultivar, respectively. These extractive water values necessitated using a physical method to improve the extractive values for date palm fruits’ phenolic components, flavonoids, and antioxidant values of the extract. Accordingly, the green use of water as a solvent in this study was assisted using three physical “factors”; time, temperature, and rotation to produce a “water bath-assisted extraction (WAE)” method. Generally, using WAE, combined with the three factors mentioned above, is typical for extracting natural products as these physical assists help to lessen the handling time and energy and to enlarge the natural extracts’ quality, quantity, and safety [45]. However, very few studies have applied pure water to extract date palm fruits with or without any physical aids. 

In the current study, the analysis of RSM (Figure 1, Figure 2, Figure 3 and Figure 4) and Table 3 reveal that the TPC of both An and Rz cultivar extracts was affected by the implementation of “factors” which gave a 2.52- and 4-fold increase in the TPC in the extract when compared with water-alone extraction, achieving 74.9% and 96.4% from the extraction value of hydro-ethanol in the An and Rz varieties, respectively. Applying the minimum of the three “factors” in An extraction resulted in no change in the amount of TPC extracted. However, increasing the time “factor” to the maximum produced a 57% increase in the TPC. Furthermore, increasing the temperature and time to the maximum resulted in a 20.3% increase in TPC related to the increase in time alone. However, when all three “factors” were applied to the maximum values, another 60.5% increase in the extract’s TPC was observed compared with the “time” and “temperature” individual increases.

Similarly, applying the minimum of the three “factors” in Rz extraction resulted in a 60% increase in the TPC extracted. Increasing the time “factor” to the maximum produced a 25% increase in the TPC. Increasing temperature and time to maximum resulted in a 35% increase in TPC compared with the increase in time alone. However, when all three “factors” were applied to the maximum values, another 48.1% increase in the extract’s TPC was observed compared with the “time” and “temperature” individual increases.

Comparably, the TFC of both An and Rz cultivar extracts gave a 2.90- and 2.68-fold increase in the TFC in the extract when compared with water-alone extraction achieving 85.7% and 92% of the extraction value of hydro-ethanol in both varieties, respectively. Applying the minimum of the three “factors” in An extraction resulted in a reduced amount of TFC extracted (0.83-fold). Increasing the time “factor” to the maximum produced a 59% increase in the TFC. Increasing temperature and time to the maximum resulted in only a 12.1% increase in TPC in relation to the increase in time alone. However, when all three “factors” were applied to the maximum values, another 96% increase in the extract’s TFC was observed compared with the “time” and “temperature” individual increases.

Similarly, applying the minimum of the three “factors” in Rz extraction resulted in nearly no change in the amount of TFC extracted. Increasing the time “factor” to the maximum produced a 34.5% increase in the TFC. Increasing both temperature and time to the maximum resulted in an 83.7% increase in TFC relative to the increase in time alone. However, when all three “factors” were applied to the maximum values, nearly no increase was observed in the extract’s TFC in relation to the “time” and “temperature” individual increases. The increase in the “rotation” factor had a very strong effect on the An cultivar; however, it was of nearly no effect in the Rz cultivar when TPC was the response. 

Regarding the antioxidant activity of the extract, the FRAP activities of both An and Rz cultivar extracts were affected by the “factors”, which gave a 2.25- and 1.46-fold increase in the FRAP activities in the extract when compared with water-alone extraction, achieving 79.3% and 84.2% of the extraction value of hydro-ethanol in the An and Rz varieties, respectively. Applying the minimum of the three “factors” in An extraction resulted in a slight reduction in the FRAP activity (0.93-fold) compared with conventional water extraction. Increasing the time “factor” to the maximum produced a 64% increase in FRAP activity. Increasing temperature and time to the maximum resulted in nearly no increase in FRAP activity compared with the increase in time alone. However, when all three “factors” were applied to the maximum values, another 35.5% increase in the extract’s TPC was observed compared with the “time” and “temperature” increases alone. For the Rz cultivar, applying the minimum of the three “factors” in Rz extraction resulted in no change in the FRAP activity of the extract. Increasing the time “factor” to the maximum produced a 20% increase in the FRAP activity. Increasing temperature and time to the maximum has resulted in no change in FRAP activity relative to the increase in time alone. However, when all three “factors” were applied to the maximum values, another 23.3% increase in the extract’s FRAP activity was observed compared with the “time” and “temperature” increases individually. Changes in the time factor affected FRAP activity in both An and Rz cultivars; however, the temperature change did not affect this, reducing activity in both cultivars. Changes in rotation moderately affected the FRAP activity in both cultivars’ extracts.

The scavenging power (ABTS) of both An and Rz cultivar extracts was influenced by the “factors” which gave a 3.81- and 4.3-fold increase in the FRAP activities in the extract when compared with water-alone extraction, achieving 113% and 121% from the extraction value of hydro-ethanol in both cultivars, respectively. Applying the minimum of the three “factors” in An extraction resulted in no change in the scavenging power (ABTS) compared with conventional water extraction. However, increasing the time “factor” to the maximum produced a 26% increase in scavenging power (ABTS). Increasing both temperature and time to the maximum has resulted in an 18.2% increase in scavenging power (ABTS) compared with the increase in time alone. However, when all three “factors” were applied to the maximum values, another 155% increase in the extract’s TPC was observed relative to the “time” and “temperature” individual increases. For the Rz cultivar, applying the minimum of the three “factors” in Rz extraction resulted in a 14% increase in the extract’s scavenging power (ABTS). Increasing the time “factor” to the maximum produced a 16.6% increase in the scavenging power (ABTS). Increasing temperature and time to the maximum has resulted in a 31.5% increase in scavenging power (ABTS) compared with the increase in time alone. However, when all three “factors” were applied to the maximum values, another 145% increase in the extract’s scavenging power (ABTS) was observed related to the “time” and “temperature” individual increases. Changes in the time factor and temperature factors did not strongly affect scavenging power (ABTS) in either An and Rz cultivar; however, the changes in rotation significantly affected the scavenging power (ABTS) in both cultivars’ extracts. 

### 4.2. The Hepatoprotective Activity of An and Rz Cultivar Extract

This study explored the hepatoprotective potentials of date palm fruit extracts in rats, specifically An and Rz, in CCl_4_-mediated hepatic injury. CCl_4_ is metalized by cytochrome P450 to the highly reactive •CCl_3_ and •CCl_3_OO radicals, which bind covalently to macromolecules to initiate a chain of events leading to membrane phospholipids’ peroxidative degradation and the accumulation of lipid-derived oxidation products causing a failure of the naturally occurring anti-oxidant defenses with subsequent hepatic injury [24]. In the current study, CCl_4_ animals exhibited reduced activity and appetite, poor nutrition, chronic diarrhea, and reduced weight gain. These findings were in agreement with Fang et al. [46] and Al-Seeni et al. [38], who reported that CCl_4_ caused a reduction in body weight. In addition, CCl_4_ resulted in the structural organization impairment of the hepatic lobules, congested hepatic portal veins, hepatocyte degeneration, inflammatory cell infiltrations, and activated Kupffer cells. MT staining revealed that the CCl_4_ group displayed hepatocyte degeneration and necrosis and increased deposition of collagen fibers in hepatic lobules, which led to the formation of a pseudo lobule that separated the lobules. Furthermore, the CCl_4_ group presented collagen fiber augmentation in the connective tissue surrounding the portal area, well-formed fibrotic bands, and abundant collagen fibers around the regenerative hepatocytes’ nodules and between the hepatocytes. On the other hand, animals co-administered with CCl_4_ alongside the date palm fruit extracts An and Rz revealed enhanced body weight gain, amended hepatic architecture, a lower leucocyte infiltration in H&E staining, ameliorated hepatocyte degeneration and necrosis, and diminished collagen fiber accumulation in MT staining. 

Liver enzymes such as ALT, AST, ALP, γGT, and LDH are hallmarks of CCl_4_ hepatotoxicity [21,38]. In the current study, CCl_4_ administration amplified ALT, AST, ALP, γGT, and LDH, indicating severe hepatic cell injury which could be attributed to tissue breakdown, permitting the escape of intracellular enzymes from the cytosol into the blood [29,47]. Administering CCl_4_ with An or Rz extracts significantly reduced the liver enzymes ALT, AST, ALP, γGT, and LDH compared with the CCl_4_-alone group. Previous studies have shown that date palm fruit extract can deter liver enzyme amplification in hepatic injuries induced by dimethoate [48], mercury [13], and radiation [49]. 

Histological examination, fibrosis grading, and hydroxyproline content in the hepatic tissue were performed to evaluate the ability of CCl_4_ to induce hepatic fibrosis. Total collagen, determined by hydroxyproline quantification as hydroxyproline, is an indicator of the collagen deposition [50]. CCl_4_-administered animals exhibited a significantly higher hydroxyproline content, which is in harmony with previous studies [51]. Meanwhile, An or Rz co-administration along with CCl_4_ resulted in a reduced level of hydroxyproline, directly reflecting a lesser amount of liver fibrosis. Bahri et al. [52] revealed that date palm fruit treatment decreased the hydroxyproline level and morphological lesions in bleomycin (BLM)-induced lung fibrosis and concluded that date palm fruit has a protective effect against BLM-induced pulmonary fibrosis because of its abundance of phenolic compounds and vitamins. Additionally, the serum alpha-fetoprotein (AFP) level was significantly elevated in the CCl_4_ group compared to the control groups. Supplementation of An or Rz with CCl_4_ resulted in a significant decline (*p* ≤ 0.05) of the serum AFP level compared with that of the untreated CCl_4_ group.

Adiponectin (ADP) is one of the most abundant adipocytokines in circulating hormones. ADP has been demonstrated to have an anti-fibrotic action in the liver through the AdipoR1 and AdipoR2 receptors, both of which are present in hepatic stellate cells (HSCs) [53]. Another theory through which ADP inhibits CCl_4_-induced liver fibrosis is the modulation of liver iNOS/NO. ADP upregulates inducible nitric oxide synthase (iNOS) in HSCs and increases nitric oxide (NO2-/NO3-) concentration in the HSC-conditioned medium [54]. In the existing study, rats administered with CCl_4_ demonstrated a significant decrease in ADP, whereas animals administered with CCl_4_ alongside An or Rz extracts exhibited a significantly increased ADP level signifying the anti-fibrotic action of date palm fruit extract. 

Nitric oxide (NO), a paracrine-acting soluble gas, possesses a wide range of biological effects, including anti-fibrotic actions [55]. In vivo studies revealed that NO has an inhibitory role in the development of liver fibrosis as it inhibits HSC proliferation and migration, promotes HSC apoptosis, downregulates the stellate cell activation marker αSMA, and suppresses collagen I gene expression [54]. The current study showed that CCl_4_ caused NO deterioration, whereas An and Rz extracts recovered the NO levels. 

The outcomes of this study revealed that liver enzyme amplification is coupled with excessive lipid peroxidation and oxidative stress, as demonstrated by augmented MDA and diminished antioxidant enzymes. Lipid peroxidation-induced damage caused an upsurge in the hepatocytes’ cellular permeability with protein leakage, including aminotransferases, into serum, indicating hepatic injury and necrosis [56]. GSH is a vital protein thiol, which coordinates the body’s defense system against oxidative stress. In addition, GSH effectively scavenges free radicals and reactive oxygen species [57]. The present study revealed that CCl_4_ injection caused a substantial reduction in GSH contents and significant depletion in the activity of phase II metabolizing enzymes such as GPX.

On the other hand, co-administration of An or Rz extracts with CCl_4_ presented a significant decline in MDA level and an escalation in the GSH and GPX. The elevation in GSH content by An and Rz observed in the current study may be due to the suppression of lipid peroxidation and protein oxidation. An earlier study showed that date fruit extract mitigated lipid peroxidation and enhanced anti-oxidant status in the liver of rats sub-chronically exposed to trichloroacetic acid [58]. Date fruits are a good source of natural antioxidants, and thus may be used as a functional food to manage oxidative stress-related disorders [59,60]. Altogether, the results of the current study demonstrated the potent effect of An and Rz extracts in alleviating CCl_4_-induced hepatic oxidative stress and lipid peroxidation. 

The inflammatory response has been reported as a critical process in CCl_4_-induced liver damage [56]. As previously described, CCl_4_-induced acute liver injury is closely associated with inflammation by elevating pro-inflammatory cytokine secretion [38,61]. Interferon-gamma (IFN-γ) effectively destroys activated HSCs (aHSCs); thus, it is a core cytokine related to the NK cell-dependent anti-fibrotic immune response [62]. In line with previous studies, the present study confirmed that the inflammatory cytokines TNF-α and NF-κB were elevated, whereas IFN-γ was diminished in the CCl_4_-induced hepatotoxicity group. By contrast, animals administered with CCl_4_ alongside An or Rz extracts exhibited decreased TNF-α and NF-κB and increased levels of IFN-γ. A previous study assessed the effect of date palm seeds’ dietary supplementation on broilers’ growth and carcass performances and revealed that date palm seeds exhibited escalations in IFN-γ, signifying the immune-stimulant constituents of the seeds [63]. Date palm fruit extract has exhibited anti-inflammatory actions in several hepatic models, such as mercury [13] or cisplatin [11] induced hepatic injury, and in non-hepatic models, such as isoproterenol-induced cardiomyopathy [64] and cisplatin-induced nephrotoxicity [65].

Similar to other macromolecules, nucleic acids are also confronted by free radicals causing oxidative DNA damage. DNA laddering is used as a fundamental feature for apoptosis. Casp-initiated DNase digests DNA at inter-nucleosomal linkers into distinct fragments of approximately 180 bp fragments. The outcomes of the DNA fragmentation assay exposed a significant increase in the DNA laddering in the CCl_4_-challenged group. Similar outcomes were reported by Alkreathy et al. [66] while studying the protective effects of *Sonchus arvensis* against CCl_4_-induced genotoxicity and DNA oxidative damage in the liver. On the other hand, co-treatment of An or Rz with CCl_4_ appreciably reduced the DNA fragmentation, indicating lesser DNA damage.

## 5. Conclusions

Two date palm fruit cultivars, An and Rz, were extracted using a newly developed green WAE method. The obtained extracts were optimized, using RSM modelling, toward the low values of “factors”: temperature, time, and rotation, and the high production of “responses”: TPC, TFC, FRAP, and ABST scavenging activity. As a result, conventional water extraction failed to extract phenolic and flavonoid compounds from the fruits’ cultivars relative to ethanol extraction (up to 25.1% and 32.6%, respectively). Similarly, conventional water extracts produced minimal antioxidant capabilities, calculated as FRAP and ABTS activities, when compared with ethanol extraction (maximum 47.7% and 29.8%). However, applying all optimization “factors”; temperature and time, and rotation, succeeded in improving the “responses” of the extraction process to reach up to 1.30, 1.23, 3.03, and 2.06-fold, respectively, in An and 2.18, 1.71, 1.11, and 2.62-fold, respectively, in Rz in relation to convectional water extraction. 

Furthermore, the optimized date palm fruit extracts of the two cultivars, An and Rz, suppressed CCl_4_-induced hepatic injury, as demonstrated by mitigating liver function parameters and improving the liver histological structure. The date palm fruit extracts exerted an anti-fibrotic effect by restoring redox balance, suppressing inflammatory cytokines, and protecting DNA structure. These results can develop the date palm fruit industry through the production of extracts enriched with phenolic and flavonoid components and/or empowered by more antioxidant functions. These findings also support the medicinal value of date palm fruit extracts in liver diseases.

## Figures and Tables

**Table 1:** Independent and dependent variables and their levels for the full factorial design of the WAE method.

Independent Variable “Factors”	Unit	Kind of Variable	Level of Variation		
			−1	0	+1
Rotational speed ^a^	(rpm)	Continuous	50	100	150
Temperature ^b^	(°C)	Continuous	30	45	60
Time	(min.)	Continuous	20	40	60
Cultivar	Type	Category	Anbara	Reziz	
**Dependent “Response” Variable**	**Abbreviation**		**Unit**		
Total phenolic content	TPC		GAE: mg/100 g fresh sample		
Total flavonoid content	TFC		QE: mg/100 g fresh sample		
Ferric reduces the antioxidant frequency	FRAP		AE: mmol/100 g fresh sample		
The ability of free radical scavenging	ABTS		Inhibition (%)		

^a^: The rotational speed is given as revolutions per minute (rpm). ^b^: The temperature of the extracted sample was measured at the end of the exposure time for each treatment.

**Table 2:** Conventional hydro-ethanol and water extraction results for the date palm fruit cultivars under investigation; Anbara (An) and Reziz (Rz). The four different “responses” were analyzed; total phenolic content (TPC), total flavonoid content (TFC), ferric reducing antioxidant power assay (FRAP), and ABTS scavenging effect (ABTS). Other “factors” of the WAE, such as “rotation” or “temperature”, were not applied, and the “time” factor was increased to 12 h.

Cultivar	Hydro-Ethanol *				Distilled Water			
	TPC **	TFC #	FRAP &	ABTS @	TPC **	TFC #	FRAP &	ABTS @
An	894 ± 18.3	63.23 ± 3.2	995 ± 46.3	78.34 ± 2.2	265 ± 10.4	18.24 ± 2.4	350 ± 24.2	23.41 ± 1.4
Rz	788 ± 59.2	50.21 ± 2.6	953 ± 62.4	74.35 ± 5.6	189 ± 22.4	16.43 ± 1.34	455 ± 22.8	20.79 ± 1.2

* Ethanol: distilled water (4:1); ** Measured as GAE: mg/100 g fresh sample; # Measured as QE: mg/100 g fresh sample; &: measured as AE: mmol/100 g fresh sample; @ Measured as inhibition (%).

**Table 3:** The ideal predicted values of the factors (rotation, temperature, and time) to obtain the maximum responses (TPC, TFC, FRAP, and ABTS) for each of the date palm fruit cultivars (Anbara and Reziz) according to the surface plot analysis (see Figure 1, Figure 2, Figure 3 and Figure 4).

Variables		Measuring Unit	Date Palm Fruit Cultivar	
			Anbara	Reziz
Affecting variables (factors)	Rotation	Rpm	103	108
	Temperature	°C	30	38.7
	Time	(min)	20.44	36.624
Response variables	TPC	(GAE: mg/100 g fresh sample)	344.322	411.295
	TFC	QE: mg/100 g fresh sample)	22.34	28.09
	FRAP	(AE: mmol/100 g fresh sample)	1061.692	506.509
	ABTS	(Scavenging effect %)	48.173	54.388

**Table 4:** Ameliorative effects of Anbara (An) or Reziz (Rz) administration on the changes in body weight of different experimental groups in CCl_4_-induced hepatotoxicity.

Animal Groups	Initial Weight (g)	Final Weight	Δ Changing in Body Weight
	Mean ± SE	Mean ± SE	Mean ± SE
Control	95.00 ± 5.00	158.50 ± 2.50	67.64 ± 6.19
An	103.33 ± 6.03	184.67 ± 6.66	79.29 ± 4.02
Rz	98.00 ± 2.29	172.00 ± 2.65	75.58 ± 1.41
CCl_4_	95.00 ± 5.00 €	103.67 ± 3.21 €	9.44 ± 2.38 €
CCl_4_ + An	102.50 ± 3.54 ₳	136.50 ± 2.12 ₳	33.39 ± 2.54 ₳
CCl_4_ + Rz	100.00 ± 5.00 ₳	136.00 ± 1.00 ₳	36.71 ± 5.84 ₳

All data are enumerated as mean ± SE, (*n* = 6). (€) describes a statistically significant correlation with the control, (₳) denotes a statistically significant correlation with the CCl_4_-induced hepatotoxicity group using one-way ANOVA after Tukey’s post-hoc test (*p* < 0.05).

**Table 5:** Ameliorative effects of An, Rz administration on the serum levels of liver enzymes including ALT, AST, ALP, γGT, and LDH in CCl_4_-induced hepatotoxicity.

Animal Group	ALT (U/L)	AST (U/L)	ALP (U/L)	γGT (U/L)	LDH (U/L)
Control	23.33 ± 2.52	163.67 ± 2.52	43.33 ± 4.04	1.53 ± 0.55	79.00 ± 4.00
An	29.50 ± 0.71	209.50 ± 0.71	51.00 ± 1.41	6.35 ± 0.50	1064.50 ± 3.54
Rz	32.60 ± 0.53	211.63 ± 0.55	53.67 ± 1.53	8.50 ± 0.46	1181.67 ± 2.08
CCl_4_	60.00 ± 3.61 €	293.00 ± 2.65 €	269.00 ± 4.00 €	21.00 ± 4.58 €	1332.67 ± 2.52 €
CCl_4_ + An	36.47 ± 0.50	250.33 ± 1.53 ₳	71.33 ± 2.52 ₳	12.33 ± 1.53 ₳	1267.33 ± 2.08 ₳
CCl_4_ + Rz	38.00 ± 1.00	273.33 ± 1.53 ₳	72.33 ± 2.52 ₳	15.00 ± 1.00 ₳	1280.67 ± 2.08 ₳

All data are enumerated as mean ± SE, (*n* = 6). (€) describes a statistically significant correlation with the control, (₳) denotes a statistically significant correlation with the CCl_4_-induced hepatotoxicity group using one-way ANOVA after Tukey’s post-hoc test (*p* < 0.05).

**Table 7:** Ameliorative effects of An or Rz administration on the serum levels of nitrosative and antioxidant enzymes in different experimental groups following CCl_4_-induced hepatotoxicity.

Animal Group	(GSH) (μmol/L)	(GPX) (μmol/L)	(MDA) (nmol/mL)	(NO) (µM/mL)
Control	57.03 ± 0.15	152.40 ± 5.36	32.53 ± 0.21	2.37 ± 0.15
An	53.50 ± 0.85	156.75 ± 3.70	122.50 ± 0.14	7.70 ± 0.28
Rz	54.23 ± 0.15	149.87 ± 2.15	69.53 ± 0.15	3.33 ± 0.04
CCl_4_	21.47 ± 0.15 €	68.47 ± 4.15 €	143.50 ± 0.10 €	2.85 ± 0.05 €
CCl_4_ + An	46.50 ± 0.20 ₳	101.30 ± 3.20 ₳	61.47 ± 0.15 ₳	4.33 ± 0.15 ₳
CCl_4_ + Rz	39.40 ± 0.10 ₳	118.70 ± 3.68 ₳	48.60 ± 0.10 ₳	4.04 ± 0.05 ₳

All data are enumerated as mean ± SE, (*n* = 6). (€) describes a statistically significant correlation with the control, (₳) denotes a statistically significant correlation with the CCl_4_-induced hepatotoxicity group using one-way ANOVA after Tukey’s post-hoc test (*p* < 0.05).

**Table 8:** Ameliorative effects of An or Rz administration on the serum levels of IFN- γ and NFκB in different experimental groups following CCl_4_-induced hepatotoxicity.

Animal Group	IFN-γ (pg/mL)	NFκB (ng/mL)	TNF-α (pg/mL)
Control	96.20 ± 0.20	68.13 ± 0.15	22.37 ± 0.15
An	98.55 ± 0.07	71.53 ± 0.15	20.73 ± 0.15
Rz	90.67 ± 0.21	73.67 ± 0.21	23.23 ± 0.03
CCl_4_	15.17 ± 0.15 €	127.85 ± 0.07 €	73.65 ± 0.35 €
CCl_4_ + An	46.73 ± 0.21 ₳	90.63 ± 0.15 ₳	27.60 ± 0.30 ₳
CCl_4_ + Rz	65.33 ± 0.21 ₳	82.33 ± 0.21 ₳	25.33 ± 0.21 ₳

All data are enumerated as mean ± SE (*n* = 6). (€) describes a statistically significant correlation with the control, (₳) denotes a statistically significant correlation with the CCl_4_-induced hepatotoxicity group using one-way ANOVA after Tukey’s post-hoc test (*p* < 0.05).

## Data Availability

The data presented in this study are openly available at Date Palm Research Center of Excellence—King Faisal University, Saudi Arabia.
